# Supplementary material for: Clinical Features and Genetic Findings of Autosomal Recessive Bestrophinopathy
Source: Genes (Basel). 2022 Jul 4;13(7):1197. doi: 10.3390/genes13071197 (PMC9320462; doi:10.3390/genes13071197)
Supplement: Supplementary file 1 [file genes-13-01197-s001.zip › genes-1780904-supplementary.pdf]

Patient No.	Fundus photography	Fundus Autofluorescence	Optical coherence tomography
1	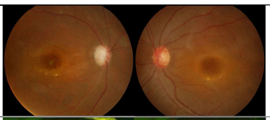	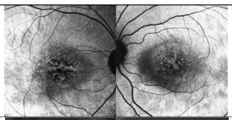	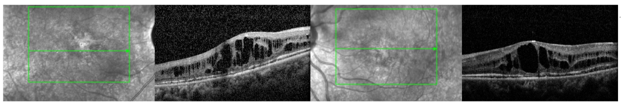
2	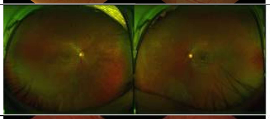	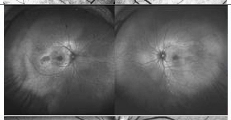	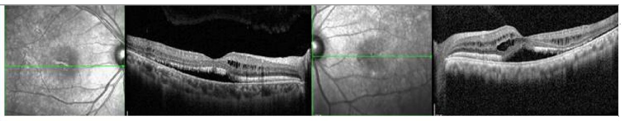
3	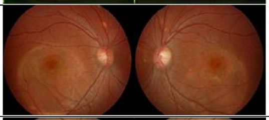	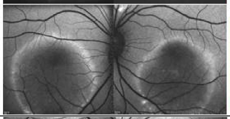	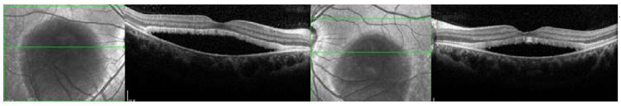
4	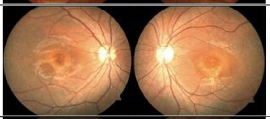	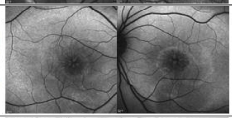	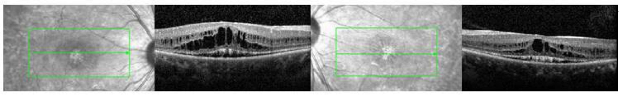
5	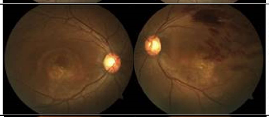	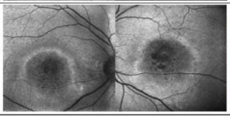	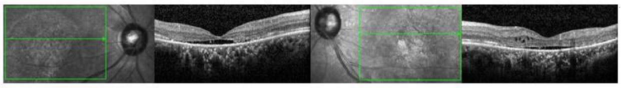
6	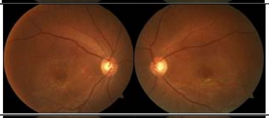	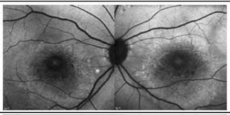	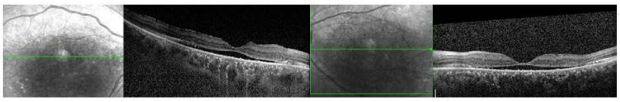
7	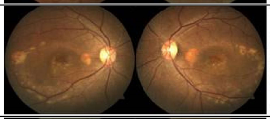	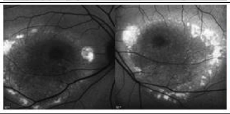	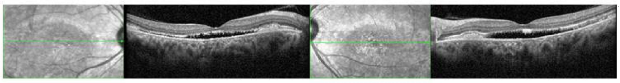
8	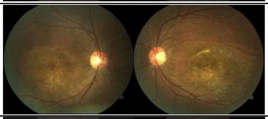	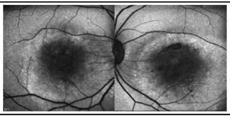	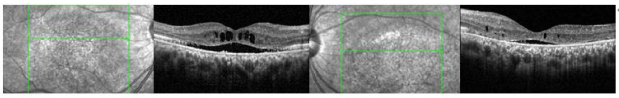
9	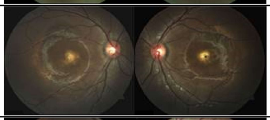	Not performed	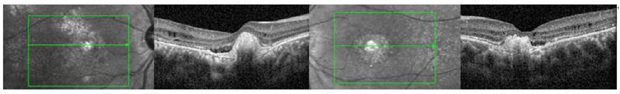
10	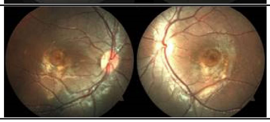	Not performed	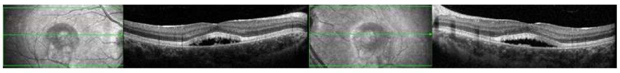
